# Effect of Functional Endoscopic Sinus Surgery on the Voice Quality among Patients with Rhinosinus Polyposis

**Published:** 2019-07

**Authors:** Mohammad Reza Majidi, Masoud Asghari, Enciye Abbaszadeh, Amir Saberi Demneh, Sohaila Hejrati

**Affiliations:** 1 *Sinus and Surgical Endoscopic Research Center, Faculty of Medicine, Mashhad University of Medical Sciences, Mashhad, Iran.*; 2 *Department of Otorhinolaryngology, Faculty of Medicine, Birjand University of Medical Sciences, Birjand, Iran. *; 3 *Department of Speech and language Rehabitation, Mashhad University of Medical Sciences, Mashhad, Iran.*; 4 *Faculty of Medicine, Semnan University of Medical Sciences, Semnan, Iran. *; 5 *Department of Speech and language Rehabitation, Faculty of Rehabitation Sciences, Mashhad University of Medical Sciences, Mashhad, Iran. *

**Keywords:** Acoustic, FESS, Nasal, Polyps, Sinus, Voice

## Abstract

**Introduction:**

Rhinosinus polyposis is associated with voice quality reduction. There has been little evidence about the efficacy of rhinosinus polyps surgery on patients' voice quality so far. The aim of the present study was to evaluate the nasality and acoustic voice changes after rhinosinus polyposis surgery.

**Materials and Methods:**

The population in this study composed of 30 eligible patients with rhinosinusitis and rhinosinus polyposiss. The functional endoscopic sinus surgery (FESS) was the therapeutic intervention. Acoustic voice parameters were jitter (%), shimmer (db), noise to harmonic ratio (NHR), and fundamental frequency (F0) for the vowels [a, o, e, aa, ie, and ou]. For nasality evaluation, the articulation of vowel [a] was examined using nasometer device. The changes regarding the patients’ voice were evaluated one day before and one month after the surgery.

**Results:**

The mean age of the participants was 41.2±14.3 years. Considering gender distribution, 20 (66.7%) subjects were men. After the operation, the nasality increased significantly from 40.8% to 74.3% (P<0.001). In addition, the findings revealed the increase of shimmer and F0 (P>0.05). On the other hand, jitter and NHR changes were insignificant.

**Conclusion:**

The findings of the current study showed that hyponasality decreased a month after the treatment of rhinosinus polyposis with FESS. However, the acoustic quality of voice had no significant changes after the surgery.

## Introduction

Patients referring to otolaryngology clinics complain about nasal and paranasal sinuses obstruction, such as reduced airflow and nasal congestion (1). Acute and chronic diseases, such as rhinosinusitis, anatomic abnormalities, allergy, and polyps, are common reasons for nasal obstruction (1,2). The type and chronicity of issues as well as treatment costs may decrease the patients’ quality of life (1). Nose and sinuses cavities in addition to respiratory role have a key role in the speech and resonance of voice (3). 

Nasal congestion and obstruction due to reduced nasal airflow cause the hyponasality of voice and poor voice quality.Rhinosinus polyps caused by the external growth of mucosa in the context of inflammation (4) have a prevalence of about 1-4% in communities (5,6). 

Rhinosinus polyp is important in some aspects since it is a chronic condition and its pathophysiology is not completely understood. It seems to have an allergic base, such as asthma and rhinosinusitis that leads to difficulty in the treatment process (7,8) and limited therapeutic alternatives (9,10). In addition to the unknown etiology, it has the recurrence rate up to 60% (11,12).

To the best knowledge of the researchers, there is little evidence regarding the effects of sinonasal polyposis treatment on voice. Previous studies also revealed conflicting results regarding the Functional Endoscopic Sinus Surgery (FESS) and its effect on the voice. Previously FESS improves voice quality and hyponasality (13,14).

 In one study, over 6 months after sinus surgery, the improvement of voice quality was not stable and reached the preoperative conditions (14). In another report over the 5 months of sinus surgery, some vowels became hyponasal and some became hypernasal (15). Acar et al. did not observe any significant difference in the acoustic properties of the voice after the implementation of FESS on the investigated patients (16). The aim of the present study was to evaluate the nasality and acoustic changes of voice before and after the rhinosinus polyp treatment with FESS. The obtained results of the current study can help to clarify the effect of this intervention on the patient’s voice quality. 

Materials and Methods 

This study was performed at Ghaem Hospital of Mashhad affiliated to Mashhad University of Medical Sciences Mashhad, Iran, during 2014-2015. The research was initiated after obtaining the approval of the ethics committee. This study was conducted on 30 patients with chronic rhinosinusitis along with rhinosinus polyps resistant to medical therapy that were subjected to FESS. The mean age of patients was 41.2±14.3 years. Regarding gender distribution, 20 (66.7%) subjects were men. 

The inclusion criteria included ages range of 18-50 years and medical treatment history of sinonasal polyps. On the other hand, the exclusion criteria were voice disorders due to pharynx and larynx as well as severe anomalies, such as cleft palate and septum deviation. The patients were assessed one day before and one month after the surgery. Based on the literature, one month after the operation, the mucosal inflammation of endoscopic surgery decreases and the first endoscopy after FESS for follow up occurs at this time.

**Surgical procedure**

Therapeutic intervention in this study was FESS with Messerklinger-Stammberger technique according to the standard protocol of general anesthesia. This method is minimally invasive with low side effects (17). In this technique, large polyps of the nasal cavity were excised, then anterior ethmoidectomy and if needed posterior ethmoidectomy and maxillary sinus antrostomy were performed. 

Sinus secretion was discharged and polyps were removed. Intervention on the frontal and sphenoid sinus was performed individually based on their involvement. Before FESS, computed tomography (CT) scan of coronal and axial sections was implemented. The Lund-Mackay score system (LMS) is the most common method to assess the intensity of sinus involvement based on the findings of CT scan findings (18). 

The obtained scores were within the range of 0-24 (clear sinuses up to completely opaque). Moreover, the rhinoscopic score for the investigation of the polyp extension against inferior turbinate was taken before FESS.

**Voice study**

To assess nasality, Nasometer device (Nasometer II- 6450) and for the acoustic voice quality Computerized Speech Lab device (CSL4500- Kompen Tax) was used one day before and one month after the FESS.

The nasometer device includes a headset that patients put it on his/her head and has a protective plate that separates the nose from the mouth. Patients in the acoustic room prolonged vowel [a] for 3 consecutive seconds, and this process was repeated twice and the mean values related to the minimum, average, and maximum of its frequency was reported. 

Then nasality of vowel [a] was calculated with the division of the nasal acoustic energy into total the nasal and oral acoustic energy in percent. To assess the voice acoustic quality, the parameters of jitter (ms), shimmer (%), harmonics to noise ratio (HNR), fundamental frequency (F0-HZ) was used for six vowels, including [â], [u], [i], [a], [e], and [o]. The fundamental frequency is the produced number of cycles per second by the vocal cords. The NHR is a noise ratio in the audio signal. The shimmer is peak-to-peak voice amplitude changes. Jitter is the period-to-period changes of voice (16).

For evaluation of the above-mentioned parameters, the patients were asked to prolong each of the above vowels for 5 sec separately. The sensitive microphone that was set 1 cm in front of the patient's mouth and nose recorded the produced sounds and transferred them to the computer. Voice acoustic benchmark was the middle 3 sec of the articulation created by the patients. In this study, the voice study tools and acoustic room for all patients were similar.

**Clinical assessment**

The scores of sinonasal polyposis symptoms, patients’ sleep quality, and fatigue sensation before and after the intervention were achieved using a questionnaire. The questionnaire was a 5-point Likert scale ranging from low to high difficulties in voice assessment procedure (1-5 scores). The questionnaire items are presented in Table 1.

**Statistical analysis**

The statistical data analysis was performed using SPSS (version 17) through the paired t-test. The quantitative data were presented as mean (±SD) and the qualitative data were reported as a percentage. P-value less than 0.05 was considered statistically significant. 

## Results

A total number of 30 patients were enrolled and followed for a month in this study. The mean age of the patients was 41.2±14.3 years. Regarding gender distribution, 20 (66.7%) patients were men. Before the FESS, the average, minimum, and maximum values of LMS were 13.1 (9.2), 0, and 24, respectively. The average, minimum, and maximum values of preoperative rhinoscopy score were 2.7 (2), 0, and 6, respectively. 

Table 1 shows the intensity of the clinical symptoms one day before and one month after the surgery. The FESS led to a significant reduction in the clinical manifestation of polyposis (except cough). Table 2 shows the changes of voice parameters one day before and one month after the FESS. After the surgery, the average and maximum values of nasality increased significantly (P<0.05). On the other hand, the minimum value decreased (P>0.05), meaning that the hyponasality decreased among the investigated patients in this study.

Table 3 indicates the results of the acoustic property of the patient's voice regarding the articulation of six vowels separately. One month after the surgery, there was a significant decrease regarding the shimmer and F0 parameters (P>0.05). Jitter decreased in some vowels and increased in some other vowels (P>0.05). On the other hand, NHR showed no significant changes (P>0.05).

**Table 1:** Changes of patients’ clinical symptoms before and after the surgery

**Symptoms**	**before**		**after**		**P**
	**Mean**	**SD**	**Mean**	**SD**	
Sneezing	1.9	1.9	1.1	1. 5	0.03
Rhinorea	2.8	1.6	1.4	1.1	<0.001
Cough	0.9	1.2	0.7	1	0.3
Post nasal drip	2.5	1.5	1.6	1.2	<0.001
Viscosity	2.9	1.5	1.4	1.5	<0.001
Ear pain	0.9	1.1	0.4	0.7	0.03
Facial pain	2.1	1.7	0.7	1	<0.001
Sleep problems	1.2	1.7	0.5	0.9	0.02
Fatigue	1	1.2	0.5	0.8	0.01
Sense of smell	3.8	1.4	1.2	1.3	<0.001
Obstruction	4	0.9	0.9	1.1	<0.001

**Table 2:** Nasality changes of vowel [ â ]

**Nasality**	**surgery**	**Mean**	**SD**	**P**
Minimum	pre	10.3	10.96	0.78
	post	11.13	12.48	
Mean	pre	46.2	22.56	0.001
	post	62.37	15.72	
Maximum	pre	81.87	26.11	0.026
	post	93.37	10.64	

**Table 3:** Changes of acoustic parameters for vowels before and after the surgery

**Parameter**		**a**		**â**		**e**		**i**		**o**		**u**	
		**Mean**	**SD**	**Mean**	**SD **	**Mean**	**SD**	**Mean**	**SD**	**Mean**	**SD**	**Mean**	**SD**
F0 (Hz)^1^*	pre	208.3	48.3	202.3	57.1	210.3	57.4	221.2	63.1	214.4	57.5	224.8	63.1
	post	194.2	59.8	196	59.8	210.0	60.8	214	64.7	208.5	60.3	217.3	64.7
Jitter (ms)*	pre	0.8	0.6	0.7	0.6	0.7	0.4	0.6	0.5	0.5	0.3	0.9	0.5
	post	0.8	0.6	0.8	.6	0.8	0.4	0.8	0.6	0.6	0.3	0.8	0.6
Shimmer (%)*	pre	2.6	1.5	2.5	1.9	2.1	1	1.9	1.3	1.4	0.6	1.7	1.3
	post	2.6	1.1	2.2	1.0	2	08	1.8	0.8	1.3	0.5	1.5	0.8
NHR (%)*	pre	0.1	0	0.1	0.1	0.1	0	0.1	0	0.1	0	0.1	0
	post	0.1	0	0.1	0.1	0.1	0	0.1	0	0.1	0	0.1	0

* No significant differences (P> 0.05).

## Discussion

According to the results of this study, FESS leads to the improvement of patients’ complications and hyponasality results from sinonasal polyposis one month after the surgery. On average, nasality improved 33.5%, However, no significant changes occurred in the acoustic parameters of voice.

There are few reports regarding the speech outcome of FESS in patients with sinonasal polyposis. the obtained results revealed that FESS can improve nasal airflow and reduce the obstruction of polyps resulting in the reduction of hyponasality. This finding was in line with the results of similar studies. Hong et al. reported hyponasality in patients with polyps increased significantly after FESS (45.7% vs 57.8%) (19). Soneghet et al. nasality improved in patients with chronic rhinosinusitis without polyps to 2.7%, 12.7%, 7.5% for the vowels of [a], [i], and [u], respectively one month after FESS. The obtained results were not significant for vowel [a]. In addition, the nasality of syllabus [ma, mi, mu] increased significantly (13).

Besides, hyponasality is another important aspect of acoustic voice. Similar to the findings of the current study, Acar et al. conducted a study on 43 patients with polyps during a 6-week follow-up after FESS voice analysis through prolonging the vowel [a]. The obtained results, revealed that FESS had no significant effect on the reduced levels of jitter, shimmer, NHR, and increased F0. Moreover, the comparison of different intensities of nasal congestion in their study showed that in severe cases of congestion, FESS led to significant reduction in shimmer index, compared to the non-sever congestion (P=0.027) (16).

In another study, the obtained results of the postoperational stage indicated that hypernasality sentence and hyponasality sentence were higher than preoperational stage. Therefore, the mean nasalance scores for vowels, nasal consonant, nasal consonant-vowel combinations increased significantly after endoscopic sinonasal surgery. After the surgery, nasalance scores were higher in all groups for all acoustic parameters. Nasalance scores did not change with widening nasal valve to use nasal strips (8).

In the present study, FESS could significantly decrease the symptoms of sinonasal polyposis after one month except for the cough. Furthermore, sleep problems, sense of smell, and daytime fatigue decreased. These patients were among those who did not respond to the treatment with medical options available for rhinosinus polyps and the surgery led to the satisfactory results. These findings are consistent with other those of other studies confirming that the patients’ signs, symptoms and quality of life improved after the nasal polyps surgery (20-23).

## Conclusion

Of the advantages of this study was the evaluation of different vowels for the better show the extent of acoustic changes. However, the limitations of this study were the small sample size and short follow-up sessions. In summary, the findings of this study indicated that among the Iranian population who have a combination of vowels and consonants in their words, FESS intervention for rhinosinus polyps led to hyponasality improvement after one month. However, it had no significant changes in the acoustic voice indices.
